# The Pharmacology and Clinical Applications of Psychedelic Medicines Within Midwifery Practice
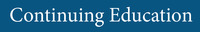


**DOI:** 10.1111/jmwh.13371

**Published:** 2022-05-06

**Authors:** Cindy A. Stein, Andrew Penn, Stephanie Van Hope, Caroline G. Dorsen, Mariavittoria Mangini

**Affiliations:** ^1^ California State University Monterey Bay School of Nursing Seaside California; ^2^ University of California at San Francisco School of Nursing San Francisco California; ^3^ Nursing & Sacred Medicine New York New York; ^4^ Division of Advanced Nursing Practice Rutgers School of Nursing Newark New Jersey; ^5^ Integral and Transpersonal Psychology Department California Institute of Integral Studies San Francisco California

**Keywords:** psychedelics, psilocybin, ketamine, MDMA, depression, PTSD, midwives

## Abstract

The research and use of psychedelic medicines to treat common mental health disorders has increased substantially in the past 2 decades. At the same time, knowledge is relatively uncommon among midwives regarding (1) the relative benefits of psychedelic‐assisted therapy, (2) best practices associated with the delivery of psychedelic‐assisted therapy, and (3) responsible integration of this potentially useful intervention into mental health treatment plans. The purpose of this review is to describe current applications of psychedelic medicines to treat common mental health disorders, to describe the current legal status of these medicines used in this context, and to explore the potential for midwifery practice in this area with further training. This article also addresses the disparities regarding LGBTQIA+ and BIPOC populations in relation to this topic and their historical exclusion from research and treatment access in this field.

## INTRODUCTION

Although Western scientists, clinicians, and individuals have been using psychedelics for decades, persons from Indigenous cultures around the globe have been using psychedelic plant medicines, such as ayahuasca, psilocybin, and peyote, for thousands of years.^1^ These medicines have historically been used for health and healing and as part of individual and community cultural and spiritual rituals.^2^ Throughout hundreds of years of colonization many Indigenous cultures held on to their longstanding plant medicine practices only to see them suddenly co‐opted by people from Western countries in the psychedelic renaissance of recent decades.^3^ In many ways, this diversion of sacred knowledge parallels the transformation of childbearing from a practice rooted in holistic healing and empowerment brought to women by midwives to the current model that often benefits a centralized medical system.

  

Continuing education (CE) is available for this article. To obtain CE online, please visit http://www.jmwhce.org. A CE form that includes the test questions is available in the print edition of this issue.

As a result, midwives have become skilled in negotiating ways to provide holistic care using intuitive, scientific, and emotional wisdom in the context of the existing health care system. Furthermore, midwives study, appreciate, attend, and facilitate transformations throughout the life span while aligning themselves with the goals of the patient and creating safe spaces for human experiences. For this reason, midwives who complete additional training in psychedelic therapy will be valuable additions to interprofessional teams that are currently researching or using these substances for the treatment of various mental health disorders. The purpose of this review is to describe current applications of psychedelic medicines to treat common mental health disorders, to describe the current legal status of these medicines used in this context, and to explore the potential for midwifery practice in this area with further training. This article also addresses the disparities regarding lesbian, gay, bisexual, transgender, non‐binary, intersex, and asexual (LGBTQIA+) and Black, Indigenous, and people of color (BIPOC) populations in relation to this topic, and their historical exclusion from research and treatment access in this field.

**QUICK POINTS**

✦ Psychedelic‐assisted therapy has emerged as a promising modality for effectively treating several mental health disorders and conditions.
✦ Midwives are qualified to participate in many advanced training programs for clinicians interested in a role within the clinical research setting or within certain clinical practice models that use psychedelic therapies.
✦ Midwives can help educate patients about existing psychedelic treatment options that may be available and provide referrals when appropriate.
✦ Efforts are needed to include BIPOC, LGBTQIA+, and other historically marginalized persons in terms of research and access to care as the field of psychedelic‐assisted therapy moves forward.

## MODERN PSYCHEDELIC‐ASSISTED THERAPY

### Historical Background

In 1957, amateur mycologist Gordon Wasson experienced psilocybin mushrooms in a traditional healing ceremony with wise woman Maria Sabina in Mexico. Wasson described the experience in a *Life Magazine* cover story, ushering in a new era of psychedelic exploration in the West.^3^ During the 1960s, hundreds of research trials were published on the clinical use of various psychedelics for treatment of depression and end‐of‐life existential distress.^4^ Although positive outcomes were typically produced with one or 2 doses of treatment, rather than the daily dosing commonly used in modern psychopharmacology (such as with antidepressants), the studies often did not have the rigor and standards required in contemporary scientific studes.^5^ However, during this research period, paradigms of care for the use of psychedelics for mental health disorders were established that continue to be foundational to current practice, such as the importance of set and setting, defined as the therapeutic qualities of the health care providers and environment, and the importance of the preparation process before the dosing experience and the subsequent integration process.^6^

Because of the public's reaction to increasing recreational use of these substances in various contexts and the accompanying societal changes, a swift reclassification of these substances to Schedule I was observed in the late 1960s to early 1970s. The result was that these medicines were labeled as having no medical use and a high potential for abuse; this closed the door on further research until determined investigators overcame prohibitive bureaucracy to reopen limited trials in the 1990s.^4^

A renaissance in psychedelic research occurred in the 2000s, with rigorous clinical trials demonstrating therapeutic effectiveness for certain conditions when provided with therapeutic support during the preparation, dosing, and integration (aftercare) processes. The research and use of psychedelic medicines to treat common mental health disorders has increased substantially in the past decade.^7^ For example, a systematic review on the use of psychedelics for treatment‐resistant depression (TRD) and anxiety yielded 7 open‐label trials that examined the effectiveness of ayahuasca, psilocybin, and lysergic acid diethylamide (LSD). Although only 7 studies representing 130 participants met inclusion criteria, every study demonstrated that “psychedelic administration caused statistically significant reductions in depression and anxiety symptoms.”^8^ ^(p^16^)^ Another systematic review of 16 articles representing 10 clinical trials examining serotonergic psychedelics and including single‐ and double‐blind placebo‐controlled randomized trials, open‐label trials, and proof‐of‐concept trials between 2000 and 2020 showed “promising early support for therapeutic effects of psychedelic compounds as adjunct to psychotherapy for mood disorders and addictions.”^9^ ^(p102)^ Current indications for use, efficacy, adverse effects, and ongoing studies of the most common psychedelic substances will be discussed in this article.

### Terminology

Although many substances may produce what can be described as psychedelic, mind‐altering, or hallucinogenic effects, these terms are too broad to describe substance classification. For the purposes of this article, the most common categorizations will be used: (1) classical psychedelics (also sometimes called serotonergic and including LSD, psilocybin, and ayahuasca),^10^ (2) entactogens (which include 3,4‐methylenedioxymethamphetamine [MDMA]),^11^ and (3) dissociatives (such as ketamine).^12^ Pharmacologically, psychedelics are known to be a heterogeneous group of compounds in chemical composition that elicit a range of subjective effects (Table 1). This article focuses on the specific medicines that are most common in current clinical trials and best positioned to become part of clinical practice in the United States. These include the serotonergic agonist psilocybin, currently under study for refractory mood disorders, refractory obsessive compulsive disorder, end‐of‐life anxiety, and tobacco and alcohol use disorders;^11^ the phenylethylamine MDMA, currently being studied for the treatment of posttraumatic stress disorder (PTSD);^13^ and ketamine and its enantiomer (mirror‐image molecule) esketamine (Spravato), cyclohexanone compounds used for the treatment of both TRD and major depressive disorder (MDD).^14^ Ayahuasca and LSD will be discussed in less depth, as these substances are not as likely to become available legally for medical treatments in the United States in the near future.

**Table 1:** Overview of Common Psychedelic Medicines Used in Research and Practice

Medicine	Indications Researched	Class	Dose, Duration of Action	Putative Mechanism of Action	Adverse and Side Effects
MDMA^34^	PTSD	Phenylethylamines	80‐120 mg orally, lasts 4‐5 h	Releaser of 5‐HT, prolactin, oxytocin Decreases overactivity of limbic (fear) brain structures, allows for processing of trauma memories and increased trust with therapist	Loss of appetite, restlessness, hypertension, insomnia, sweating, trismus, anxiety
Psilocybin^10^, ^67^	MDD/TRD Tobacco use Alcohol use disorder Anxiety, depression, demoralization related to life‐threatening illness	SerotonergicClassical 5‐HT2 agonist Tryptamines	10‐25 mg orally, lasts 4‐6 h	Potent 5‐HT2A agonist Appears to decrease activity in key brain networks related to depression	Anxiety, nausea, sweating, restlessness, headache
Ketamine/ esketamine^68^, ^69^	MDDTRD PTSD PPD	DissociativeCyclohexanone NMDA antagonist	54‐88 mg intranasally (esketamine) 0.5 mg/kg IV (ketamine) Lasts one h	Glutamate (NMDA) receptor antagonist Leads to rapid neuronal growth and branching	Nausea, dissociation, derealization, blurry vision, drowsiness, laryngospasm (rare)
Ayahuasca^47^	Substance use disorders Anxiety Depression	SerotonergicClassical 5‐HT2 agonist, combined with MAO inhibitor	Very variable dosing depending on preparation; peaks at 60 and 120 min after ingestion, lasts about 4 h	Works as an agonist at the cortical 5‐HT receptors decrease default mode network activity	Nausea, vomiting, visual disturbances, loss of sense of time
LSD^70^	Alcohol use disorders Anxiety	SerotonergicClassical 5‐HT2 agonist Ergoline	0.5 and 2 mcg/kg Lasts up to 12 h	Elicits its effect through agonist activity in the 5‐HT2A receptor and possibly the 5‐HT2C and 5‐HT1A receptors	Time distortion, hallucination, emotional distress

Abbreviations: 5‐HT, serotonin; IV, intravenously; LSD, lysergic acid diethylamide; MAO, monoamine oxidase; MDMA, 3,4‐methylenedioxymethamphetamine; MDD, major depressive disorder; NMDA, *N*‐methyl‐d‐aspartate; PPD, postpartum depression; PTSD, posttraumatic stress disorder; TRD, treatment‐resistant depression.

### Psilocybin

#### Pharmacology

Psilocybin is a naturally occurring compound in the genus *Psilocybe* mushroom and is a prodrug, rapidly dephosphorylating into psilocin, which bears a structural similarity to serotonin (5‐HT). As such, its primary targets are postsynaptic 5‐HT2A receptors, which are densely expressed in key limbic and cortical areas.^15^, ^16^, ^17^ Activation of these receptors leads to downstream inhibition of key information routing hubs in the brain. This results in decreased activity of the default mode network (thought to be overstable in depression, which inhibits the activation of the externally focused task positive network) and transiently reorganizes the routing of the brain's internal communication. This may serve as a reset in a brain that has become rigidly fixed in a particular behavioral and emotional phenotype such as that seen in depression.^18^

#### Clinical Applications and Research

Psilocybin specifically has been studied for the treatment of major depression,^19^, ^20^ TRD,^21^ end‐of‐life anxiety,^22^, ^23^ tobacco cessation,^24^ and alcohol cessation.^25^ Symptoms of depression in various studies are often immediately and significantly reduced after one dose, with effects lasting months and, for some study participants, years.^26^ An open‐label study using 2 doses of psilocybin 7 days apart (10 mg and then 25 mg) assessed efficacy of treating TRD with the Quick Inventory of Depressive Symptomatology, the Snaith‐Hamilton Pleasure Scale, the Beck Depression Inventory, and the supplementary State‐Trait Anxiety Inventory trait scale. Depression ratings significantly improved at the one‐week and 3‐month post‐treatment points.^21^ A follow‐up study found that the antidepressant results remained significant at 6 months after treatment, giving hope that psilocybin could become a useful tool in treating depression.^27^ These results are similar to a randomized double‐blind crossover trial of 51 patients with cancer who had symptoms of depression and anxiety and were randomized to either very low (placebo‐like), low (1 or 3 mg/70 kg), or high (22 or 30 mg/70 kg) dose psilocybin with 5 weeks between sessions. The largest decreases in observed and self‐reported symptoms of depression and anxiety were seen in the high‐dose group with sustained results for almost 80% of participants at 6 months.^22^ Various studies that examined the effect of psilocybin on mood disorders (including major depression, end‐of‐life anxiety, and TRD) reported a positive correlation between mystical‐type experiences and antidepressant response.^28^ In one study, mystical experience was measured on the Pahnke‐Richards Mystical Experience Questionnaire, which measures subjective experiences of (1) internal unity, (2) external unity, (3) transcendence of time and space, (4) ineffability and paradoxicality, (5) sense of sacredness, (6) noetic quality, and (7) deeply felt positive mood. The study included 36 participants, with 67% of participants in this study describing their psilocybin experience as one of the most meaningful experiences of their lives.^29^ Prominent themes in qualitative descriptive accounts of participants’ psilocybin experiences include “exalted feelings of joy, bliss, and love; embodiment; ineffability; alterations to identity; a movement from feelings of separateness to interconnectedness; experiences of transient psychological distress; the appearance of loved ones as guiding spirits; and sharing the experience with loved ones post‐treatment.”^30^ ^(p354)^ No serious adverse events have been reported in any trial to date, only transient, expected side effects (see Table 1). However, in a study of psilocybin users in the community, 2.6% of participants self‐reported behaving in a physically aggressive or violent manner, and 2.7% reported receiving medical help.^31^ This contrast highlights the importance of the therapeutic environment and therapeutic relationship to mitigate or manage the physical and psychological risks. As a Schedule I drug, psilocybin is not currently available for medical use outside of clinical trials, but it has been granted a Breakthrough Therapy designation by the US Food and Drug Administration (FDA), fast‐tracking its development and indicating that the drug treats a serious or life‐threatening condition and offers substantial improvement over available therapies.^32^ The first movement toward legal clinical use outside of the research setting in the United States occurred in November 2020 when the people of Oregon passed Ballot Measure 109, which established regulations and rules by the Oregon Health Authority to implement the use of psilocybin by licensed providers beginning in 2023.^33^

### MDMA

#### Pharmacology

MDMA is sometimes called an entactogen, as opposed to a classical psychedelic, for its ability to create feelings of connectedness with oneself and others. MDMA is a potent releaser of presynaptic 5‐HT, norepinephrine, prolactin, and oxytocin.^34^ MDMA is usually administered orally; its effects begin 30 to 45 minutes after ingestion, peak at about 90 to 120 minutes, and last for 4 to 6 hours.^34^ It appears to decrease the overactivity of the amygdala (the brain's alarm system) found in PTSD while increasing activity in the prefrontal cortex, where traumatic memories are examined and recontextualized during the therapeutic process.^32^

#### Clinical Applications and Research

MDMA has been demonstrated as an effective treatment for chronic PTSD when used in tandem with psychotherapy during dosing sessions.^13^, ^35^ The pooled results of 6 clinical trials sponsored by the Multidisciplinary Association for Psychedelic Studies (MAPS) yield the most comprehensive data on MDMA in the treatment of PTSD to date. These randomized, double‐blind, controlled clinical trials completed between 2004 and 2017 showed that with oral doses of MDMA (75 mg and 125 mg doses) given in 2 separate 8‐hour sessions one month apart, 72 treatment participants experienced significantly reduced PTSD symptoms compared with 31 participants who received placebo or control doses of 0 to 40 mg. PTSD symptoms were reduced and sustained in the long term by 54.2% in the MDMA dosing groups compared with a 22.6% improvement in the control group.^13^ A recent phase 3 trial demonstrated that 67% of participants in the treatment group versus 32% participants in the control group with refractory PTSD no longer met PTSD diagnostic criteria after treatment using the Clinician‐Administered PTSD Scale for the *Diagnostic and Statistical Manual of Mental Disorders*, Fifth Edition (CAPS‐5), the most widely accepted instrument for quantifying PTSD symptoms.^36^ The studies used a protocol that included 3 nonmedicine therapy preparatory sessions before treatment began and 3 to 4 integration sessions after dosing sessions. Although chronic recreational use of MDMA or ecstasy has been associated with deficits in memory, cognitive processing, sleep, and psychiatric status,^37^ these deficits have not been noted in clinical trials, and very few adverse events have been reported within trials (see Table 1).^32^ Within the context of current MAPS‐sponsored clinical trials, the following exclusion criteria apply: current psychotic disorder , bipolar disorder 1, current borderline personality disorder, eating disorder with active purging, pregnancy, and lactation. MDMA was granted FDA Breakthrough Therapy designation in 2017 and is currently in phase 3 clinical trials.^13^

### Ketamine

#### Pharmacology

Ketamine is an analog of phencyclidine and is a glutamate receptor antagonist, developed in the 1960s for the induction of anesthesia.^14^ It is often called a dissociative because of early reports of side effects noted by patients undergoing anesthesia who experienced unexpected, intense, dissociative or hallucinatory experiences.^14^ Its mechanism of action is complex, binding to phencyclidine site 2 in the *N*‐methyl‐d‐aspartate receptor on glutamatergic neurons, which begins a cascade of events including brain‐derived neurotrophic factor release and mammalian target of rapamycin disinhibition, leading to rapid neuronal dendritic growth and branching. It is this rapid, within hours to days, neurogenesis that is thought to account for the antidepressant effects of racemic ketamine and its left stereoisomer, esketamine.^38^ In the clinical setting, ketamine is most often given intravenously, by intramuscular injections, intranasally (esketamine), or sublingually.

#### Clinical Applications and Research

Ketamine is a Schedule III drug, and in addition to its use in anesthesia, it is able to be legally prescribed off‐label for TRD and MDD, providing immediate and dramatic, although short‐acting, improvement in symptoms at subanesthetic doses.^39^ In a recent double‐blind, randomized, placebo‐controlled study, 35 patients with TRD were randomized to one of 3 infusion groups (0.5 mg/kg, n = 12; 0.2 mg/kg, n = 11; or normal saline, n = 12). The Hamilton Depression Rating Scale and the Montgomery‐Åsberg Depression Rating Scale were completed by participants at 40 and 240 minutes after infusion and then on days 2 to 7 and day 14. Researchers found that the higher dosing (0.5 mg/kg) led to a significant reduction in depression and suicidal symptoms but not in anxiety distress symptoms. The lower dose (0.2 mg/kg) did not show a rapid reduction in depression symptoms and suicidal ideation.^40^ A systematic review of 6 randomized, placebo‐controlled double‐blind clinical trials completed between 1990 and 2013 with participants diagnosed with unipolar and bipolar MDD diagnoses showed that ketamine can rapidly improve depressive symptoms both one day and 7 days after infusion.^41^ Another systematic review of 28 primary studies demonstrated that a single infusion of ketamine reduced clinical depression symptoms within 24 hours and that the benefits were sustained for between 6.8 and 30 days for the 4 single‐infusion studies that measured relapse times.^42^ Esketamine (Spravato), an intranasally administered enantiomer, or mirror image, of the ketamine molecule, is approved by the FDA under a Risk Evaluation and Mitigation Strategy for the treatment of TRD and MDD in patients concurrently using antidepressant therapies.^43^

More recent studies have examined the efficacy of ketamine in relation to postpartum depression. One randomized, double‐blind placebo‐controlled trial assessed rates of postpartum depression among women with low‐risk pregnancies who had not received a previous diagnosis of depression by a psychiatrist. The women received either intravenous ketamine or placebo (saline) during a scheduled cesarean. The researchers found significantly higher scores on the Edinburgh Postnatal Depression Scale (EPDS) among the placebo group (22.6%) at one week postpartum than among the group that received 0.25 mg/kg of ketamine (13.1%), with a *P* value of .029 and a number of 165 in both groups.^44^ However, depressive symptoms were not significantly different between the intervention and control groups by 2 weeks postpartum.

Another randomized controlled trial measured EPDS scores preoperatively and at 2 and 4 weeks postoperatively. Women received either 1 to 2 mg/kg of sodium thiopental and 0.5 mg/kg of ketamine or just 3 to 5 mg/kg of sodium thiopental to induce anesthesia for their scheduled cesareans.^45^ Depression scores between the intervention and control groups were not significantly different preoperatively. At 2 weeks postoperatively, the control group had an increase in the mean EPDS score to 14.34, although it was not significant, whereas the intervention group showed a significant decrease in the mean EPDS scores to 11.82 using a *P* value less than 0.001. At 4 weeks postoperatively, the control group had a decrease in EPDS score to a mean of 13.09, which was a significant difference from the 2‐week postoperative mark, but not from the original EPDS mean scores. The intervention group had a decreased to a mean score of 10.84, which was significantly different from the preoperative scores, but not significantly different compared with the 2‐week scores for that group.^45^ Essentially, although both groups showed significant improvements in depression scores at various times, the trending decrease happened at different points, warranting further study to examine why.

The dissociative and hallucinatory effects of ketamine have previously been interpreted as an unpleasant side effect. However, in recent decades a new approach to the use of ketamine at various doses and modes of administration, in tandem with various types of therapy models, has embraced these consciousness‐altering elements of ketamine as therapeutic.^14^

Because the use of ketamine to treat depression is an off‐label application, rigorous safety data from clinical trials are not available. Chronic, frequent abuse of ketamine has been associated with impairments in memory, aspects of executive function, reduced psychological well‐being, and bladder cystitis.^46^ For this reason, some clinicians prefer supervised in‐office dosing of intramuscular or intravenous ketamine as opposed to oral at‐home dosing to reduce the risk of dependence or abuse.^12^ Esketamine (Spravato) is subject to an FDA‐mandated Risk Evaluation and Mitigation Strategy, further limiting risk of diversion or misuse.

### Ayahuasca

#### Pharmacology

Ayahuasca is used by Indigenous peoples in parts of the Amazon jungle for certain rituals and therapeutic reasons. It is a beta‐carboline– and dimethyltryptamine‐rich hallucinogenic botanical mixture made into a brew by combining bark of the *Banisteriopsis caapi* vine—which contains beta‐carboline alkaloids and serves as a monoamine oxidase inhibitor in the gut—and the leaves of the *Psychotria viridis* bush. Both of these supply the hallucinogen *N*,*N*‐dimethyltryptamine (DMT).^47^ DMT is both a derivative and an analog of tryptamine and is structurally similar to psilocybin. It may decrease overstable default mode network activity and has the potential to treat symptoms of depression and substance use disorders.^48^ Like psilocybin and LSD, ayahuasca also acts as an agonist at the cortical 5‐HT receptors. The effect of ayahuasca typically begins approximately 40 minutes following ingestion, peaks at 60 to 120 minutes, and lasts about 4 hours. Common experiences include visual imagery, nausea and vomiting, contact with the spiritual world, inability to communicate verbally, and loss of sense of time.^47^

#### Clinical Applications

There were 2 systematic review of clinical trials done between 2016 and 2020 that comprised 9 studies—5 with human subjects and 4 that were preclinical. Both reviews presented data to show optimistic results in the reduction of substance use and anxiety with ayahuasca. However, the reviews included studies that were low to moderate in study design quality because of the limited amount of research on this medicine, therefore it is not possible to draw any definitive conclusions about efficacy.^49^ The botanical admixture of ayahuasca and its status as a sacred Indigenous plant medicine make it unlikely to become available soon for legal medicinal purposes in the United States.

### LSD

#### Pharmacology

The psychoactive effects of LSD were accidentally discovered in 1943 and later studied in the 1950s and 1960s as a psychiatric drug.^50^ Like psilocybin, it seems that LSD's primarily action is to create effect through agonist activity in the 5‐HT2A receptor and possibly the 5‐HT2C and 5‐HT1A receptors.^5^ Dosing is generally between 0.5 and 2 mcg/kg and can cause time distortion, hallucinations, and increased emotional expression for up to 12 hours.^5^

#### Clinical Applications

A systematic review of LSD studies in psychiatry between 1950 and 2019 described 11 randomized controlled trials and found mixed results. Although there are historical data to support that LSD may be useful specifically in the treatment of alcohol use disorders, the variability of study designs has made it difficult to interpret the full effects in several studies, including those looking at anxiety and depression.^5^ Another issue is the inability to create double‐blind clinical trials because of the obvious hallucinatory and observable behavioral differences once LSD is ingested.

### Microdosing

Microdosing of psychedelics is the practice of taking a very small subperceptual dose of a psychedelic, most commonly psilocybin or LSD, on a frequent basis such as 3 days per week, with the aim of treating depression or enhancing mood, creativity, and focus. Popularized by online communities, recent studies have found significant expectancy effects.^51^ In a single randomized, placebo‐controlled trial,^52^ the effects of microdoses of psychedelics had no significant difference compared with placebo. Additionally, Szigeti et al showed that participants who expected to be taking a microdose but were actually taking a placebo also experienced benefit, indicating that expectancy bias likely significantly influenced reported microdosing outcomes.

## IMPLICATIONS FOR MIDWIFERY PRACTICE

Midwifery is a profession that requires efficient interpersonal communication, humanistic qualities such as compassion, respect, and patience, and the ability to synthesize scientific and evidence‐based knowledge into practice. Midwives who obtain advanced training in psychedelic‐assisted therapy are therefore uniquely prepared to contribute to interprofessional clinical research and treatment. These skills are considered ideal assets of the therapists, guides (those who sit with patients as they undergo a medicine session), health care providers, educators, and advocates who work collaboratively in the field. The precise competencies identified for psychedelic therapists or guides are (1) an empathic abiding presence, (2) trust enhancement, (3) spiritual transpersonal intelligence, (4) knowledge of the physical and psychological effects of psychedelics, (5) therapist self‐awareness and ethical integrity, and (6) proficiency in complementary modalities.^6^ Penn et al (2021) made the astute observation that

Nurses are skilled in holding space as patients endure challenging events in real time and for prolonged periods, whether that be during childbirth, a sudden illness, an anxiety attack, or the time surrounding death. This skill translates well to being able to sit with a patient undergoing a therapeutic psychedelic experience, allowing space for whatever arises at physical, emotional, mental, or spiritual levels.^53^ ^(p39)^

Midwives already have many of these skills and are experienced in spending long periods of time *with woman* while using support skills such as therapeutic touch, interpersonal communication, and intuition while clients experience enormous life transitions. Midwives are also often the first or only health care provider to encounter people when they are experiencing perinatal depression or anxiety, birth trauma, eating disorders, addiction issues, sexual assault, or general depression and anxiety. Therefore, with added training, midwives could screen, identify, and refer people for psychedelic‐assisted therapy when appropriate.

Psychedelic‐assisted therapy and research are still in the early phases of evolution, and the role of midwives within this setting remains entirely undefined. However, midwives are qualified to enroll in many specialized advanced training programs to be able to contribute to both research and clinical aspects of this emerging specialty, attend conferences and continuing education programs, and network with relevant organizations that offer research and seminar resources (see Table 2).

**Table 2:** Programs, Institutions, and Organizations Where Midwives Can Seek Information and Advanced Training on Psychedelic‐Assisted Research and Therapy

Organization or School	Training Opportunities, Conferences, and Organizations
California Institute of Integral Studies	Certificate program in Psychedelic‐Assisted Therapy and Research https://www.ciis.edu/research‐centers/center‐for‐psychedelic‐therapies‐and‐research
Naropa Institute	Certificate in Psychedelic‐Assisted Therapies https://www.naropa.edu/academics/extended‐campus/psychedelic‐assisted‐therapies‐certificate/
Multidisciplinary Association for Psychedelic Studies (MAPS)	Training in theory, skills, and practice of 3,4‐methylenedioxymethamphetamine (MDMA)–assisted therapy https://maps.org/2016/01/29/mdma‐therapy‐training‐program/
Fluence	Education in psychedelic‐assisted psychotherapy and integration https://www.fluencetraining.com/
Polaris Insight	Didactic and experiential trainings and retreats for clinicians on ketamine‐assisted therapy in the clinical setting https://www.polarisinsight.com/training‐retreats/
Salt City Psychedelic Therapy and Research	Psychedelic therapy training program https://www.scptr.org/psychedelic‐therapy‐training‐program‐
Psychedelic Research and Therapy Institute	Ketamine‐assisted therapy trainings https://pratigroup.org/
The Ketamine Training Center	Ketamine‐specific training workshops https://theketaminetrainingcenter.com/overview/
Horizons NYC	Live and digital forums; classes and films that look at psychedelic drugs and plant medicines in science, medicine, culture, and spirituality; hosts the largest and longest‐running annual gathering of the psychedelic community in the world https://horizonspbc.com/newyork
Interdisciplinary Conference on Psychedelics	https://icpr2020.net/
Chacruna Institute for Psychedelic Plant Medicines	https://chacruna.net/
Usona Institute	Research organization that supports and conducts preclinical and clinical research on the therapeutic effects of psilocybin and other consciousness‐expanding medicines https://www.usonainstitute.org/about/
Organization of Psychedelic and Entheogenic Nurses (OpeNurses)	Represents nurses, at all levels of training, who work with patients using therapeutic psychedelic medicines https://www.openurses.org/
Sana Symposium	Official annual meeting of the Psychedelic Medicine Association https://www.sanasymposium.com/

## THE PSYCHEDELIC‐ASSISTED THERAPY PROCESS

Effective and safe psychedelic therapy requires extensive preparation of the participant, all members of the care team, and the environment in which the therapy will take place. Much like within the birthing environment, participants’ experiences during psychedelic therapy can be influenced by the context in which it takes place. This preparation is described as the *set* (mindset) and *setting* (environment) and includes the intentions, attitudes, and behaviors of those present, as well as the physical or built environment and how these factors affect the patient response to psychedelic medicines.^6^, ^54^

Patients seeking for psychedelic‐assisted therapy must meet criteria either to participate in an authorized clinical trial, or for ketamine treatment by a licensed clinician. Ketamine treatment options exist within health systems and in private practices across the United States. At the time of this writing, there are currently clinical trials examining MDMA‐assisted therapy for PTSD, psilocybin‐facilitated therapy for major depression (alone or comorbid with Parkinson's disease or cancer diagnosis), obsessive compulsive disorder, early dementia, alcohol use disorder, cocaine use disorder, eating disorders, bipolar 2 disorder, and chronic headaches (see Table 3). Regardless of the specific medicine used, all psychedelic‐assisted therapy generally follows a similar process. The therapist or guide, who may be a health care provider, clergy member, or other mental health clinician with advanced training in psychedelic‐assisted therapy, first establishes the set and setting during preparatory sessions with the participant. During these sessions of varying number and duration, the patient and therapist determine individual patient intentions, discuss expectations, and review the medicine session process. Recent studies such as those that MAPS has conducted have 6 to 8 hours of preparatory sessions in the weeks before the medicine is administered in order for the patient to receive the most benefit.^55^, ^56^

**Table 3:** Clinical Trials Currently Enrolling Participants in Psychedelic Therapy Research

Medicine	Clinical Indications Being Examined	Where to Find Information
MDMA: MAPS (global)	PTSD	https://mdmaptsd.org/
Psilocybin (global)	TRD, MDD, smoking cessation, headaches (cluster and migraine), eating disorders, substance use disorders, depression and anxiety in patients with Parkinson's	https://clinicaltrials.gov/ct2/home
Psilocybin: Usona Institute (United States)	Depression	https://www.usonainstitute.org/about/#pat‐car
Psilocybin: Johns Hopkins (United States)	Smoking cessation, depression, Alzheimer's, depression with alcohol use disorder, anorexia nervosa	https://hopkinspsychedelic.org/
Ketamine	Mood disorders with suicidal ideation, MDD, TRD, PPD, prenatal depression, depression in patients with Parkinson's	https://clinicaltrials.gov/ct2/home

Abbreviations: MAPS, Multidisciplinary Association for Psychedelic Studies; MDD, major depressive disorder; MDMA, 3,4‐methylenedioxymethamphetamine; PPD, postpartum depression; PTSD, posttraumatic stress disorder; TRD, treatment‐resistant depression.

Creating the most beneficial experience for participants for the actual medicine or dosing session(s) also typically includes a process whereby the environment is intentionally arranged so that soft light, natural elements, and music generate a calm, private, and comfortable ambiance for healing and self‐exploration to take place. The setting can have a significant influence on the impact of the medicine experience, create an optimal environment for each individual, and limit negative outcomes.^54^, ^57^ Although there are obvious stylistic difference in how health care providers approach set and setting, the most common features include the use of eyeshades,^58^ a couch or other comfortable area to recline on, sheets and blankets that can be layered as needed, visual art, and a musical playlist that is curated specifically to correlate with the onset, peak, and recession actions of the medicine being used.^55^ Music plays a critical part of the therapeutic process as it can influence and enhance the subjective emotional experience of a participant.^59^, ^60^, ^61^

The session typically begins with settling the participant comfortably into the calm setting, observing initial vital signs, answering any remaining questions, and reiterating what to expect and what will be the plan of care for the session. Two therapists, or guides, typically stay with the patient during the entire duration of the medicine session and provide support and monitoring to maintain safety standards and offer encouragement and reassurance to the participant. Psychedelic therapists use a wide array of skills during this phase and may facilitate commencing a session using guided breathing techniques or reciting rituals or prayers that the participant feels comfortable with or has requested. Once the medicine has been administered, support is provided through a broad range of methods depending on the individual needs of the participant and the safety guidelines that are in place. This may include ongoing measurements of vital signs such as blood pressure, therapeutic touch as needed and agreed upon beforehand, quiet observation and transcribing or using audio to record anything the participant verbalizes, visual cues to give reassurance if the participant removes the eyeshades, and verbal prompts and encouragement for the participant to explore inward.^62^

The period directly after the medicine session and in the weeks following serves as a window during which behavior and thought patterns can be discussed and examined in what is called the integration phase.^7^ Integration of the experience may take place in a single session shortly after the peak action of the medicine, over one or more sessions during the days and weeks following the session, or between multiple sessions in a treatment plan. The goal of integration is to help participants process the medicine session and translate that experience into meaningful life changes. Integration offers feedback, skills, and activities to promote healing and can consist of various therapeutic modalities including art, journaling, somatic practices, and different types of talk therapy.^62^

## PSYCHEDELIC MEDICINE IN THE CONTEXT OF SOCIAL JUSTICE

Increasing interest in psychedelic medicine research and use to maximize physical and mental health, treat addiction, heal trauma, and in end‐of‐life care has led to the urgent need to consider numerous ethical and social justice issues. Specifically, and similarly to the profession of modern midwifery, questions have arisen related to cultural appropriation, Indigenous rights, and racial, economic, and LGBTQIA+ equity. Little has been written about the inclusion of LGBTQIA+ persons in psychedelic research or in the possible application of psychedelic medicine to address the special health care needs of these communities. Efforts to increase inclusion of LGBTQIA+ persons in psychedelic research are needed, as well as research that centers the potential impact that psychedelics might have on existing health and social disparities in LGBTQIA+ communities. Similarly, and much like in midwifery, where the lack of racial and ethnic diversity in clinicians and leaders is prevalent,^63^, ^64^, ^65^ racial justice issues in psychedelic medicine are further complicated by minimal diversity among psychedelic researchers.^63^ Research also suggests that BIPOC people may have decreased access to psychedelic care and are underrepresented in clinical trials.^63^, ^65^ The main socioeconomic barrier to access is that psychedelic‐assisted therapy is not currently covered by insurance and, outside of clinical trials, can cost from hundreds to thousands of dollars per treatment either for ketamine‐based therapies in the United States or by requiring costly travel to other countries for care using substances that are not currently legal here. Additionally, ethical questions exist regarding cultural appropriation and exclusion of Indigenous persons in the planning and execution of psychedelic research and practice^3^ and the potential for financial exploitation as these plant medicines gain in popularity and are decriminalized in some regions of the United States.^66^

## CONCLUSION

Psychedelic‐assisted therapy has many known and emerging applications that may benefit patients who receive care from midwives. These applications include common mental health disorders and conditions like depression, anxiety, PTSD, and substance use disorders. Midwives should have basic knowledge of these therapies as an increasing number of people are using them both within the United States and globally. Obtaining advanced training to better understand the pharmacology and physiology and demonstrate proper use of psychedelic medicines within the health care arena can be of great benefit to midwives and the patients whom they serve.

## CONFLICT OF INTEREST

The authors have no conflicts of interest to disclose.
